# Editorial: Plasticity and Reconstruction of Neural Network in Brain Injury

**DOI:** 10.3389/fncel.2021.710499

**Published:** 2021-06-22

**Authors:** Pengyue Zhang, Roxanne Ilagan, Yulong Bai, Xiangjian Zhang, Yunping Deng, Yuchuan Ding

**Affiliations:** ^1^College of Acupuncture, Tuina and Rehabilitation, Yunnan University of Traditional Chinese Medicine, Kunming, China; ^2^Department of Neurosurgery, Wayne State University School of Medicine, Detroit, MI, United States; ^3^Department of Rehabilitation Medicine, Huashan Hospital Affiliated to Fudan University, Shanghai, China; ^4^Department of Neurology, Second Hospital of Hebei Medical University, Shijiazhuang, China; ^5^University of Tennessee Health Science Center (UTHSC), Memphis, TN, United States

**Keywords:** plasticity, neural network, brain injury, reconstruction, synaptogenesis

The brain is a complex organ which controls all our functions such as movements, balance, thoughts, memory, emotions, circadian clock, and hormone secretion. One of its characteristics is to be very plastic where by plasticity we mean its ability to modify its connections in order to adapt to new internal or external stimuli including traumatic injury, and ischemic or hemorrhagic stroke. Brain injuries lead to the loss of brain parenchyma through necrosis, apoptosis and autophagic cell death of neural cell (Liao et al., 2020). Regardless of the etiology, the breakdown of the entire neural network is the direct result of brain injury and the immediate cause of dysfunction. The exact symptoms of brain disorders depend on the damaged area of the brain. Damages in the language centers or hippocampus result in speech dysfunction or spatial learning and memory disorders. Damages in the motorsensory cortex result in motor and sensory dysfunction of limbs despite normal structure of the spine, peripheral nervous system, bones, and muscles. Thus, the repair and reconstruction of the injured neural network is the ultimate goal for the treatment of brain injury. A thorough clarification of the cellular and molecular mechanisms of plasticity in brain injury and of the mechanisms of neural network reconstruction is a necessary endeavor in the search for new therapeutic targets.

The plasticity found in brain injury differs from the regeneration seen in spinal cord injury: it occurs in a complex environment that includes neural stem cells, injured neurons (surviving bodies and dead axons or synapses), denervated intact neurons, and numerous glial cells. Thus, reconstruction of the neural network after brain injury is affected by multiple cellular and molecular mechanisms, and the synergy among these factors contributes to the reconstruction and recovery of function.

The microenvironment is the functional foundation of the neural network both in the normal and injured brain. The components of the microenvironment include a variety of cell types (i.e., normal and dysfunctional neurons, activated endogenous neural stem cells, astrocytes and microglia in different activated states), blood capillaries, glial scars, secreted factors (such as free radicals, neuroinflammatory factors, and neurotrophic factor), and the extracellular matrix (see the findings from Roll et al. in this topic). Some components are beneficial to the plasticity of the injured brain, but others are damaging. In this topic, Lei et al. found that NF-κB activated miR-146a-5p and induced oxidative stress and pyroptosis via TIGAR in a hippocampal neuronal cell model of AD; knockdown of NF-κB markedly attenuated oxidative stress and pyroptosis which protected neurons. Results from Hou et al. suggested that pharmacological inhibition of CSF1R caused acute activation of the microglia, promoted inflammatory response, and aggravated neuronal degeneration; this in turn caused loss of dendritic spines and behavioral deficits after transient global cerebral ischemia.

In addition, the newly formatted microvessels in the injured region are an important component of the microenvironment for neural plasticity and repair—damage activates the proliferation of endothelial cells and vascular remodeling through up-regulating the expression of a group of angiogenic factors including vascular endothelial growth factor (VEGF), Ang1/2, and their receptor Tie2. In addition, arteriogenesis (i.e. collateral artery growth, a process in which pre-existing collateral arterioles transform into functional collateral arteries) plays an important role in maintaining cerebral blood flow and improving the microenvironment in ischemic regions after brain injury (Sugiyama et al., 2011). Thus, in order to create a suitable microenvironment for neural plasticity and repair, we must promote the favorable factors and inhibit the adverse factors simultaneously.

Another key component to the reconstruction and repair of the neural network is endogenous neural stem cells (NSCs) (Jinno, 2021). Under external stimuli, such as brain injury, these NSCs from the subventricular zone (SVZ) and dentate gyrus (DG) can proliferate, migrate, and differentiate into neurons, oligodendrocytes, and astrocytes. The new neurons can incorporate into network circuitry, the oligodendrocytes can repair myelin sheaths, and the astrocytes can support, protect, and nourish neural networks in reconstruction. Because of the low immunogenicity and good histocompatibility as well as self-renewal and multi-directional differentiation potential, exogenous NSC transplantation has been used as a treatment for various neurological diseases. Numerous preclinical studies demonstrated that transplantation of exogenous NSCs could complement or replace damaged tissues and improve function (Chen et al., 2016; Zhang et al., 2019). Findings from Bai et al. demonstrated that bone marrow mesenchymal stem cells (BM-MSCs) could differentiate into mature and functional nerve cells, promote nerve regeneration, and improve functional recovery.

Creating the neural connection is a critical step for the reconstruction of the neural network and it requires axon formation, synaptogenesis, and myelination. Axons can sprout from new neurons derived from neural stem cells, injured neurons with surviving bodies and dead axons or synapses, and existing normal neurons located outside of the damaged zone. Axon formation goes through multiple steps including neuritogenesis, axon growth, axon maturation, axon branching, and pruning. The whole process is regulated by multiple mechanisms involved in astrocyte regulation (Fossati et al., 2020). Axon formation creates neuronal polarity and is a prerequisite to synaptogenesis and neural connection. Afterwards, the synapse begins to form between neuron terminals and the dendrites or body of another neuron, and the neural network is reconstituted (Zheng, 2020). The reconstituted neural network then needs to further mature and stabilize under the stimulation of rehabilitative training as well as activities of daily living. At this point, the injured neural network has been reconstructed and the dysfunction has been recovered wholly or partly.

Although the injury can initiate the spontaneous reconstruction of neural networks, the regenerative process is not sufficient for the complete reconstruction of the neural network and full recovery of function. Thus, therapies that stimulate the endogenous neural plasticity and reconstruction of the neural network are critical. Clinical and preclinical trials have determined that neuromodulation techniques such as repetitive transcranial magnetic stimulation (rTMS), transcranial direct current stimulation (tDCS), intermittent theta-burst stimulation (iTBS), and deep brain stimulation (DBS) can promote neural plasticity and improve recovery of function. However, the parameters of treatment such as the dose, frequency, location, and timing after stroke need further study. Ding et al. demonstrated that iTBS in the motor cortex (M1) did not enhance the accuracy or facilitate neuroplasticity after brain-computer interface (BCI) training.

Several other treatments and their effects on neural plasticity have also been studied. Physical exercise and enriched environment have been proven to improve neural plasticity after brain injury–the involved mechanism includes modulation of several mechanisms such as inhibition of the acute inflammatory response as well as enhancement of angiogenesis and synaptogenesis (Zhang et al., 2013, 2020; Hannan, 2014). In addition, Chinese herbs extracts have been shown to promote the reconstruction of the neural network and improve neurobehavioral deficits. Results from Hu et al. showed that notoginsenoside R1 derived from Panax notoginseng promoted neural repair through regulation of Nav proteins in an Alzheimer Disease model. Kang et al. demonstrated that Cannabidiol extracted from cannabis rescued mitochondrial dysfunction induced by MPP+ by activating autophagy. These results suggested that Cannabidiol could be used for the treatment of Parkinson's disease. In addition, electro-acupuncture was identified to improve the functional recovery in ischemic stroke through suppression of neuronal autophagy and apoptosis and by promoting the differentiation of endogenous neural stem cell (eNSC) (see results from Wang et al., Xing et al., and Zhang et al. in this topic).

Although numerous therapeutic methods have been proven effective in animal and preclinical experiments, the clinical application of these treatments needs to be further investigated. A comprehensive therapeutic regimen including a variety of therapeutic methods could contribute to the plasticity and reconstruction of the neural network and requires further research.

## Author Contributions

PZ, YDi, YB, XZ, and YDe discussed the contents of this topic. PZ written the editor article. YDi, YB, XZ, YDe, and RI revised the article. All authors contributed to the article and approved the submitted version.

## Conflict of Interest

The authors declare that the research was conducted in the absence of any commercial or financial relationships that could be construed as a potential conflict of interest.
